# Urinary Markers of Tubular Injury and Renal Fibrosis in Patients with Type 2 Diabetes and Different Phenotypes of Chronic Kidney Disease

**DOI:** 10.3390/life13020343

**Published:** 2023-01-27

**Authors:** Anton I. Korbut, Vyacheslav V. Romanov, Vadim V. Klimontov

**Affiliations:** Research Institute of Clinical and Experimental Lymphology—Branch of the Institute of Cytology and Genetics Siberian Branch of Russian Academy of Sciences (RICEL—Branch of IC&G SB RAS), 630060 Novosibirsk, Russia

**Keywords:** type 2 diabetes, diabetic kidney disease, renal fibrosis, tubular injury, albuminuria

## Abstract

This study assessed the urinary excretion of markers and mediators of tubular injury and renal fibrosis in patients with type 2 diabetes (T2D) and non-albuminuric and albuminuric patterns of chronic kidney disease (CKD). One hundred and forty patients with long-term T2D and different patterns of CKD and twenty non-diabetic individuals were included. Urinary retinol-binding protein 4 (RBP-4), glutathione-S-transferase α_1_ and π (GST-α_1_ and GST-π), transforming growth factor β (TGF-β), type I and type IV collagen (Col1 and Col4), bone morphogenic protein 7 (BMP-7), and hepatocyte growth factor (HGF) were assessed by ELISA. Patients with T2D demonstrated increased urinary excretion of RBP-4, GST-π, Col4, BMP-7, and HGF (all *p* < 0.05 vs. control). The excretion of RBP-4, GST-π, Col1, and Col4 was increased in patients with elevated albumin-to-creatinine ratio (UACR; all *p* < 0.05 vs. control), while BMP-7 and HGF were increased innormoalbuminuric patients also (*p* < 0.05). Urinary RBP-4, GST-α_1_, Col1, Col4, and HGF correlated positively with UACR; meanwhile, no correlations with glomerular filtration rate were found. The results demonstrate that elevated urinary excretions of the markers of tubular injury (RBP-4, GST-π) and renal fibrosis (Col1, Col4), as well as HGF, an antifibrotic regulator, are associated with the albuminuric pattern of CKD in subjects with T2D.

## 1. Introduction

An increased prevalence of diabetes around the world and changes in the management of the disease have transformed the epidemiology and clinical course of chronic kidney disease (CKD) in recent years. A number of studies have documented an increasing proportion of patients with diabetes developing a reduction in renal function without a preceding or concomitant increase in albuminuria [1,2,3,4].

It was demonstrated that albuminuric and non-albuminuric CKD patterns differ in their pathogenic mechanisms, risk factors, and clinical course [2,5,6].Albuminuric CKD turned out to be associated with classical glomerular injury, whereas tubulointerstitial and vascular changes may be more prominent in the non-albuminuric CKD phenotype [7]. We have demonstrated recently that elevated urinary excretion of nephrin and podocin, the components of podocyte slit diaphragm, is associated with albuminuria in patients with type 2 diabetes (T2D). Meanwhile, elevated excretion of WAP four-disulfide core domain protein 2 (WFDC2), a marker of tubulointerstitial fibrosis, is associated with a decrease in renal function in these patients [5].

The study of markers of glomerular and tubular dysfunction and renal fibrosis is considered a promising approach to the diagnosis and prognosis of diabetic kidney diseases [8,9,10]. We hypothesized that urinary markers of tubular dysfunction, as well as factors involved in renal fibrosis, may respond differently in albuminuric and non-albuminuric phenotypes of diabetic CKD. Therefore, we aimed to assess the urinary excretion of some tubular markers and fibrotic and antifibrotic mediators in patients with T2D and non-albuminuric and albuminuric CKD.

## 2. Materials and Methods

### 2.1. Study Design

This observational, single-center, and cross-sectional study included adult participants with long-term T2D. Men and women with the disease duration of more than 10 years since diagnosis were enrolled. The following set of exclusion criteria was established: end-stage renal disease, verified non-diabetic CKD, current urinary tract infection, ketoacidosis or hyperosmolar state at the time of the survey, malignant neoplasms, inflammatory or autoimmune diseases in the medical history, and high-protein diet.To reduce the risk of misclassification of CKD variants, we also excluded patients receiving antihyperglycemic drugs with established antialbuminuric effects: dipeptidyl peptidase 4 (DPP-4) inhibitors, glucagon-like receptor 1 (GLP-1) receptor agonists and/or sodium-glucose cotransporter 2 (SGLT-2) inhibitors for three months prior to inclusion.

Patients were recruited from the institutional database(2012–2018) as described previously [5,11].We formed 4 groups of patients, 35 people each, based on the estimated glomerular filtration rate (eGFR) and urinary albumin-to-creatinine ratio (UACR). We considered eGFR below 60 mL/min/1.73 m^2^ as a sign of decreased renal function (DRF) and UACR ≥ 3.0 mg/mmol as elevated albuminuria (EA). The values of eGFR ≥ 60 mL/min/1.73 m^2^ and UACR < 3.0 mg/mmol were referred to as normal renal function (NRF) and normal albuminuria (NA), respectively. Therefore, NRF/NA, DRF/NA, NRF/EA, and DRF/EA groups were identified. The classification principle is shown in Table 1.

We also included 20 individuals without diabetes, obesity, or cardiovascular diseases as healthy control. The exclusion criteria were the same for control and diabetic subjects.

We assessed urinary excretion of retinol-binding protein 4 (RBP-4), glutation-S-transferase α_1_ (GST-α_1_), and glutation-S-transferase π (GST-π) as markers of tubular injury [12,13,14]. Among these markers, GST-α_1_ and GST-π are considered to be site-specific. GST-α_1_ is located predominantly in the proximal convoluted tubules. Additionally, low levels of GSTα have been detected in the loop of Henle [15]. Urinary GST-π is attributed to distal tubules and collecting ducts [13,14,16].

We measured urinary excretion of transforming growth factor β (TGF-β), a principalfibrotic mediator in the diabetic kidney [17,18]. In addition, urinary Col1 andCol4 were assessed as markers of fibrosis [16,19,20]. We also estimated urinary concentrations of two growth factors with antifibrotic potential: bone morphogenic protein 7 (BMP-7) [21,22] and hepatic growth factor (HGF) [23,24].

### 2.2. Methods

Routine laboratory measurements, including glycated hemoglobin A_1C_ (HbA_1C_), serum creatinine, UACR, and urinary excretion of total protein, were performed on AU480 Chemical Analyzer (Beckman Coulter, Brea, CA, USA). The eGFR was calculated by the CKD-EPI formula (2009).

The concentrationsof RBP-4, GST-α_1,_ GST-π, TGF-β, Col1, Col4, BMP-7, and HGF were assessed in the morning urine samples by ELISA. The commercially available kits: SEA929Hu for RBP-4; SEA609Hu for GST-α_1_; SEB090Hu for GST-π; SEU124Hu for TGF-β;HEA571Hu for Col1; HEA180Hu for Col4; SEA799Hu for BMP-7;and EA047Hu for HGF(Cloud Clone Corp., Katy, TX, USA) were used in this study. The activation reagent IS044 (Cloud Clone Corp., Katy, TX, USA) was applied additionally for the measurement of TGF-β concentration. None of the received results were out of the upper limit of the range defined by the kit manufacturer. The results which were below the lower limit of the range defined by the manufacturer were set as zero. The results were adjusted to the urinary creatinine.

### 2.3. Statistical Procedures

The sample size was preliminary calculated with parameters: α = 0.05 and 1 − β = 80% [25,26].The minimum number of participants was defined as 30 per group.

The Statistica 13.0 software package (Dell, Round Rock, TX, USA) was used for statistical procedures. The normality was tested by the Shapiro-Wilk test. As most of the quantitative variables were not distributed normally, Kruskal-Wallis H tests were used to assess the differences between the groups. Intergroup differences in discrete parameters were analyzed using the χ^2^ test. A difference was considered significant if the *p*-value was below 0.05.

Spearman rank correlation analysis was used to assess the associations between urinary markers and other parameters. The associations of markers with CKD patterns were checked in logistic regression models. The markers were adjusted on age, sex, body mass index (BMI), diabetes duration, and glycated hemoglobin (HbA_1C_). The contributor in regression analysis was defined as significant if the standard deviation of the coefficient beta did not exceed the coefficient beta and the *p*-value was less than 0.05.

## 3. Results

### 3.1. Clinical Characteristics of Patients

One hundred and forty individuals with T2D were included in the study. Clinical characteristics of patient groups are presented in Table 2. Patients with different CKD phenotypes did not differ significantly in age, sex, BMI, diabetes duration, and treatment modalities (all *p* > 0.05). Patients with elevated albuminuria and normal renal function (NRF/EA group) had higher HbA_1C_ as compared with those without CKD (NRF/NA group; *p* = 0.04).

### 3.2. Urinary Excretion of Tubular Markers

**Figure 1:**
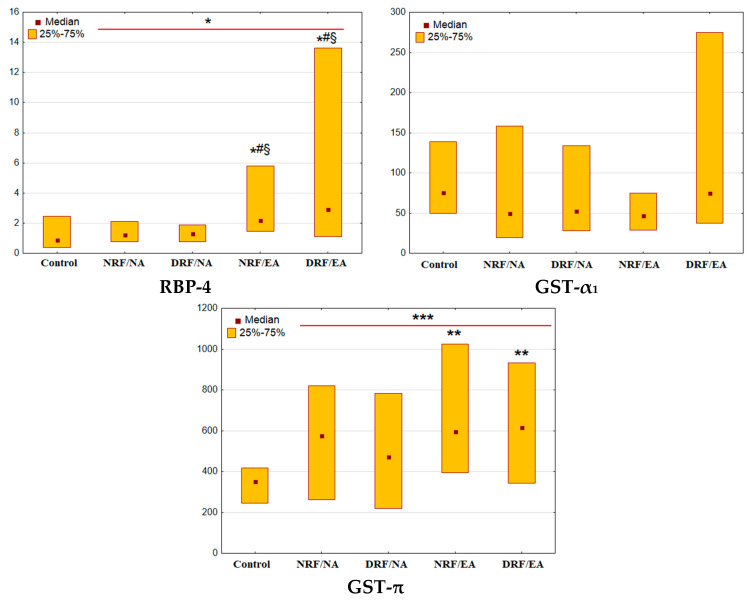
Urinary excretion of tubular markers in patients with T2D and different patterns of CKD. RBP-4, retinol-binding protein 4 (μg/mmol); GST-α_1_, glutation-S-transferase α_1_ (ng/mmol); GST-π, glutation-S-transferase π (ng/mmol); * *p* < 0.05, ** *p* < 0.01, *** *p* < 0.001 vs. control group, ^#^ *p* < 0.05 vs. NRF/NA; ^§^ *p* < 0.05 vs. DRF/NA (Mann-Whitney U-test or Kruskal-Wallis H-test for comparison of two groups or multiple comparisons, respectively).

Patients with T2D had higher urinary excretion of RBP-4 and GST-π than control individuals (*p* = 0.03 and *p* < 0.001, respectively, Figure 1). However, no differences were found in GST-α_1_ excretion.

The changes in the RBP-4 and GST-π excretion were more prominent in albuminuric groups (NRF/EA group: *p* = 0.01 and *p* = 0.004, respectively; DRF/EA group: *p* = 0.02 and GST-π: *p* = 0.005). The increase in RBP-4 excretion in both NRF/EA and DRF/EA groups was significant in comparison with NRF/NA and DRF/NA groups (NRF/EA vs. NRF/NA, *p* = 0.02; DRF/EA vs. NRF/EA, *p* = 0.01;DRF/EA vs. NRF/NA, *p* = 0.03;DRF/EA vs. DRF/NA, *p* = 0.02).

Urinary RBP-4 and GST-π, but not GST-α_1_, correlated positively with UACR (r = 0.55, *p* < 0.001; r = 0.27, *p* = 0.003; r = 0.07, *p* = 0.44, respectively). No correlations were found between tubular markers and eGFR (RBP-4 and GST-α_1_: r = −0.08, *p* = 0.33; GST-π: r = − 0.10, *p* = 0.26).

### 3.3. Urinary Excretion of TGF-β, Col1, and Col4

**Figure 2:**
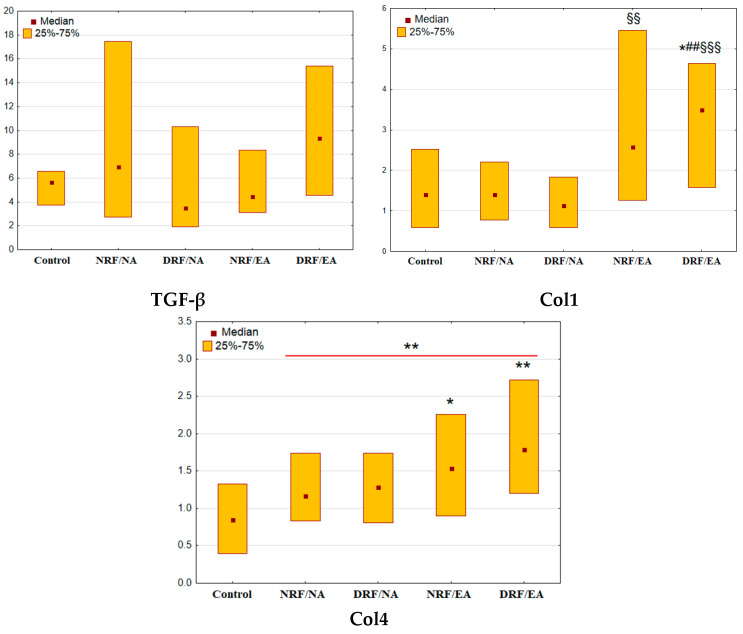
Urinary excretion of TGF-β, Col1 andCol4 in patients with T2D and different patterns of CKD. Col1, type I collagen (pg/mmol); Col4, type IV collagen (ng/mmol); TGF-β, transforming growth factor β (ng/mmol); * *p* < 0.05, ** *p* < 0.01 vs. control group, ## *p* < 0.01 vs. NRF/NA group, ^§§^ *p* < 0.01, ^§§§^ *p* < 0.001 vs. DRF/NA (Mann-Whitney U-test or Kruskal-Wallis H-test for comparison of two groups or multiple comparisons, respectively).

Patients with T2D demonstrated no significant changes in TGF-β and Col1 excretion (*p* = 0.56and *p* = 0.11 vs. control, respectively, Figure 2). Diabetic groups were not different from each other in the urinary TGF-β (all *p* > 0.05). Nonetheless, the patients with elevated albuminuria were characterized by higher levels of urinary Col1 as compared with other groups of the participants (NRF/EA: *p* = 0.006 vs. DRF/NA; DRF/EA: *p* = 0.02 vs. control, *p* = 0.008 vs. NRF/NA, *p* < 0.001 vs. DRF/NA).

Patients with T2D as a whole group had elevated Col4 excretion (*p* = 0.002, Figure 2). Diabetic groups with elevated albuminuria, but not those with normal UACR, demonstrated increased Col4 excretion when compared to control (NRF/EA: *p* = 0.03; DRF/EA: *p* = 0.002; NRF/NA: *p* = 0.45; DRF/NA: *p* = 0.48).

The excretion of Col1 and Col4, but not TGF-β, correlated positively with UACR (Col1: r = 0.49, *p* < 0.001; Col4: r = 0.34, *p* < 0.001; TGF-β: r = 0.17, *p* = 0.06). No correlations with eGFR were found (Col1: r = −0.05, *p* = 0.50; Col4: r = −0.09, *p* = 0.28; TGF-β: r = −0.06, *p* = 0.49).

### 3.4. Urinary Excretion of BMP-7 and HGF

**Figure 3:**
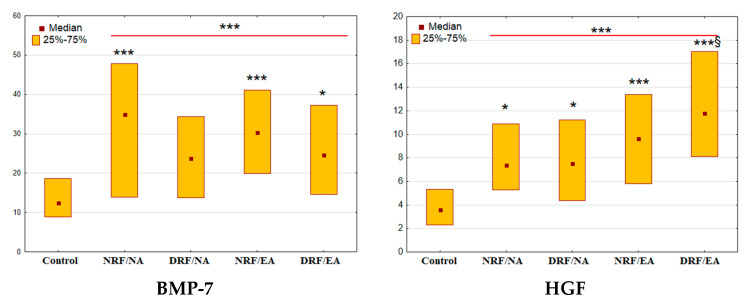
Urinary excretion of BMP-7 and HGF in patients with T2D and different patterns of CKD. BMP-7, bone morphogenetic protein 7, μg/mmol; HGF, hepatocyte growth factor, ng/mmol. * *p*< 0.05, *** *p* < 0.001 vs. control group, § *p* < 0.05 vs. DRF/NA group (Mann-Whitney U-test or Kruskal-Wallis H-test for comparison of two groups or multiple comparisons, respectively).

The excretion of BMP-7 and HGF in patients with T2D was increased significantly compared to the control (both *p* < 0.001, Figure 3).

Among subjects with diabetes, the elevation of BMP-7 was significant in NFR/NA, NRF/EA, and DRF/EA groups (*p* < 0.001, *p* < 0.001, and *p* = 0.03 vs. control, respectively). Meantime, the excretion of HGF was increased in all diabetic groups (NRF/NA: *p* = 0.02; DRF/NA: *p* = 0.03, NRF/EA and DRF/EA: both *p* < 0.001). Additionally, DRF/EA group demonstrated higher urinary excretion of HGF when compared to the DRF/NA group (*p* = 0.02).

The excretion of HGF correlated positively with UACR (r = 0.33, *p* < 0.001) but not with eGFR (r = −0.10, *p* = 0.26). The excretion of BMP-7 did not correlate with UACR (r = 0.05, *p =* 0.57) and demonstrated a weak correlation with eGFR (r = 0.20, *p =* 0.02).

### 3.5. Urinary Markers in Multivariate Models

In logistic regression models, RBP-4, Col1,and HGF were associated with increased albuminuria(UACR ≥ 3.0 mg/mmol) after adjustment for age, sex, BMI, duration of diabetes, and HbA_1C_ (Table 3). No independent associations were found between studied markers and declined renal function (eGFR < 60 mL/min/1.73 m^2^).

Urinary Col1 was associated with the DRF/EA phenotype after adjustment for age, sex, duration of diabetes, and HbA_1C_. RBP-4 was associated with both albuminuric phenotypes (NRF/EA and DRF/EA) after adjustment for these factors(Table 4).However, we could not build any reliable model with assessed renal markers for non-albuminuric CKD.

## 4. Discussion

In this study, we tested the hypothesis that urinary tubular markers, as well as factors involved in renal fibrosis, may respond differently in albuminuric and non-albuminuric phenotypes of diabetic CKD. We demonstrated that urinary excretion of RBP-4, GST-π,and Col4 was increased in patients with T2D and that change was more prominent in patients with albuminuria independently from the renal function decline. Additionally, we found an elevation of urinary Col1 in patients with T2D and albuminuria. We also found an elevation of the urinary excretion of BMP-7 and HGF in patients with T2D. The elevation of BMP-7 was more reliable in patients with preserved renal function, while the excretion of HGF was higher in patients with albuminuria.

### 4.1. Tubular Markers

In our patients, urinary RBP-4 demonstrated a clear association with albuminuric CKD.RBP-4, a member of the lipocalin family, is the major transport protein for retinol in circulation. The expression of RBP-4 is highest in the liver and adipose tissue. Besides, RBP-4 mRNA was also detected in the kidney [27]. An increased serum concentration of RBP-4 with a positive correlation with serum creatinine and cystatin C was reported in patients with proteinuric CKD [28]. Inverse relations between eGFR and serum RBP-4 were found in Chronic Kidney Disease: Determinants of Progression and Cardiovascular Risk (PROGREDIR)study [29].

The associations between urinary excretion RBP-4 and UACR were found previously [30]. The elevated urinary RBP-4 is assumed to be a consequence of disrupted reabsorption of RBP-4 in the proximal renal tubule by the megalin-cubilin receptor complex [27]. RBP is a ligand of megalin [31]. Megalin gene knock-out was associated with decreased urinary reabsorption of albumin and RBP in mice with streptozotocin-induced diabetes and *Akita* mice [32]. The elevated expression of megalin was found in normoalbuminuric streptozotocin-induced diabetic rats and in immortalized human proximal tubular cells (HK-2) [33]. These data are consistent with our results demonstrating elevated excretion of RBP-4 in T2D patients with albuminuric CKD patterns.

The predictive value of urinary RBP-4 was assessed in some previous studies. Urinary RBP-4 was associated with rapid renal function decline in kidney transplant recipients [34]. High urinary level of RBP-4 was an independent predictor of progressive CKD during a 24-month follow-up in severe non-alcoholic fatty liver disease(NAFLD) patients with hypertension [35].

GSTs are a superfamily of ubiquitous detoxification isoenzymes that conjugate substrates to reduced glutathione [15]. Our study did not reveal the elevation of urinary excretion of GST-α_1_ in patients with T2D. Groups of participants with albuminuric and non-albuminuric CKD did not demonstrate changes in urinary excretion of GST-α_1_ either. Similar results were obtained in patients with type 1 diabetes (T1D) [13]. The study with a larger number of participants (N = 457) with T2D, T1D, and other types of diabetes and prediabetes also failed to demonstrate the difference in urinary excretion of GST-α between individuals with different grades of albuminuria [16].

We found increased urinary excretion of GST-π in patients with T2D; this increase was associated with albuminuria regardless of renal function. Increased urinary excretion of GST-π can be a result of the enzyme release from damaged tubular cells into urine [13,14,16]. Thus, elevated urinary GST-π may indicate damage of distal tubules in patients with long-term T2D and increased albuminuria.

### 4.2. TGF-β, Col1, and Col4

TGF-β is a key signaling molecule in the development of renal fibrosis [17,18,21]. Increased urinary excretion of TGF-β may reflect an increase inTGF-β production in the kidney [36]. The elevated excretion of TGF-β was described previously in patients with T1D [37,38] and T2D [39].

In our study, we failed to find any differences in urinary TGF-β between control subjects and patients with diabetes, as well as between patients with albuminuric and non-albuminuric CKD. The decreased renal production and urinary excretion of TGF-β were observed previously under an ACE inhibitor administration in rats with subtotal renal ablation [40]. Similarly, treatment with losartan reduced urinary excretion of TGF-β in patients with T2D [41,42]. In our study, the vast majority of patients were treated with renin-angiotensin system blockers. This may explain the lack of intergroup differences in the excretion of this factor.

Col1 is considered to be a component of normal kidney interstitium and glomerular basement membrane. The deposition of Col1 in these renal compartments is increased intubulo interstitial fibrosis. Glomerulosclerosis is associated with de novo expression of Col1in the mesangial matrix [19]. In a large multicenter prospective study (N = 1767, including 935 patients with diabetes),associations of urinary excretion of Col1 α1-chain with baseline eGFR, stage of CKD, and eGFR slope per year were demonstrated [20]. Our study did not reveal the association between eGFR and urinary Col1, while we found a strong association with albuminuria. In a previous cross-sectional study, an association between serum concentrations of carboxy-terminal propeptide of type I procollagen and albuminuria was found in patients with T2D [43].

Col4 is a major component of the glomerular and tubular basal membrane and mesangial matrix [18]. An association between urinary excretion of Col4 and albuminuria was revealed in some previous studies that enrolled patients with diabetes [44,45,46].These findings are in agreement with our results demonstrating increased urinary Col4 excretion in T2D patients with an albuminuric pattern of CKD. At the same time, we found no association between the excretion of Col4 and eGFR. The lack of association between urinary Col4 and eGFR was also noted in a cross-sectional study involving1554 individuals with diabetes [47] and in a prospective study with 1067 participants without diabetes in Japan [48]. However, high urinary excretion of Col4 was recognized as a risk factor for progressive renal function decline in the non-diabetic population [48].

Col1 and Col4 are considered as molecules associated with kidney fibrosis [19]. In turn, renal fibrosis is a determinant of CKD progression [49]. In our study, the urinary excretion of Col1 and Col4 was higher in albuminuric patterns of CKD. The data correspond to the results of studies demonstrating that patients with non-albuminuric CKD have a lower risk of CKD progression compared to those with elevated albuminuria [50,51].

### 4.3. BMP-7 and HGF

BMP-7, a member of the TGF-β superfamily, demonstrated an anti-fibrotic effect in diabetic nephropathy [52]. It was found that overexpression of renal BMP-7 can inhibit TGF-β/Smad3 signaling and protect the kidney from TGF-β-mediated injury [53,54]. BMP-7 represses albumin-induced chemokine synthesis in tubular epithelial cells through the destabilization of NF-κB-inducing kinase [55]. In this study, we revealed hyperexcretion of BMP-7 in patients with T2D, mainly in those with preserved renal function. Therefore, it can be assumed that the increase in BMP-7 synthesis in diabetic kidneys is a protective event.

In experimental models of diabetic nephropathy, a decreased expression of BMP-7 was found in mesangial cells and renal tissues [56,57]. A high level of BMP-7 expression in proximal tubules and podocytes was revealed at the early stages of human diabetic nephropathy; however, low expression of BMP-7 was found at advanced stages of the disease [58]. The increased expression of BMP-7 was noted under pitavastatin treatment in podocyte cell culture [59] and streptozotocin-induced diabetes in Wistar rats [60]. In our study, a substantial proportion of participants with T2D were treated with statins. Therefore, the effect of treatment on factor synthesis cannot be ruled out.

HGF is a multifunctional cytokine that plays an important role in development, regeneration, and tissue repair [23,54]. In the diabetic kidney, HGF targets glomerular cells and reduces both glomerular and tubulointerstitial fibrosis [23,61].Another possible mechanism involves the activation of autophagy-lysosome pathwaysin podocytes [55,62,63].Unexpectedly, we found elevated excretion of HGF in patients with T2D, especially in those with albuminuric CKD. We also found an association between urinary HGF and albuminuria in the logistic regression model. Therefore, the role of HGF in the pathogenesis of diabetic kidney diseases needs further research.

### 4.4. Why do Urinary Markers Play Differently in Albuminuric and Non-Albuminuric CKD?

Various hypotheses can be proposed to explain the preferential association of the studied renal markers with the albuminuric phenotype of CKD.

Albuminuria is considered to be the result of an increased glomerular filter permeability and disrupted tubular absorption of albumin [64]. Similar to RBP-4, urinary albumin is reabsorbed by megalin in tubules [65,66]. As a result, elevated urinary excretion of the studied molecules could be a consequence of higher glomerular barrier permeability or disrupted tubular reabsorption of these proteins. Disrupted reabsorption is the most probable pathway for elevated urinary excretion of RBP-4 [27]. However, higher serum RBP-4 was described in patients with T2D previously [66].

Some molecules can enter the urine from damaged kidney structures. This could be a probable explanation for elevated excretion of GST-π, a marker of a tubular injury [13,14,16], Col1, a component of the mesangial matrix in glomerulosclerosis, glomerular basal membrane and interstitium [19], and Col4, a component of the glomerular and tubular basal membrane [16,19].

One could speculate that altered bone metabolism may contribute to the elevation of urinary Col1 excretion in albuminuric patients with diabetesand CKD. However, previously, aninverse association of UACR with bone turnover markers, such as procollagen type 1 N-terminal propeptide (P1NP) and β-C-terminal telopeptide of type I collagen (β-CTx),was reported [67].

Finally, activation of the synthesis of the studied molecules in the kidney may increase their urinary concentrations. Elevated production of BMP-7 in renal tissues was demonstrated previously at the early stages of diabetic nephropathy [58] and under statin treatment [59,60]. Increased renal expression of Col1 and Col4 has been recognized as a sign of diabetic nephropathy [19]. The association between renal expression and urinary excretion of Col4 was described [68].

### 4.5. Limitations of the Study

Our study is not without limitations. Due to the variability in albuminuria and eGFR, some patients could be misclassified when divided into groups. Some intergroup differences may not have been found due to the limited sample size. The cross-sectional design is another obvious limitation. Future prospective studies are needed to elucidate the value of urinary RBP-4, GST-π, Col1, Col4, BMP-7, and HGF as predictors of albuminuric and non-albuminuric CKD in T2D subjects.

## 5. Conclusions

Patients with T2D have elevated urinary excretion of markers of tubular injury (RBP-4, GST-π), renal fibrosis (Col1, Col4), and antifibrotic growth factors (BMP-7, HGF). In these patients, urinary RBP-4, GST-π, Col1, Col4, and HGF areassociated with albuminuria independently from renal function. The identification of markers of non-albuminuric CKD in T2D remains a challenge for future research.

## Figures and Tables

**Table 1:** Patient groups.

Group	eGFR	UACR	N
NRF/NA	≥60 mL/min/1.73 m^2^	<3.0 mg/mmol	35
DRF/NA	<60 mL/min/1.73 m^2^	≥3.0 mg/mmol	35
NRF/EA	≥60 mL/min/1.73 m^2^	<3.0 mg/mmol	35
DRF/EA	<60 mL/min/1.73 m^2^	≥3.0 mg/mmol	35

eGFR, estimated glomerular filtration rate; UACR, urinary albumin-to-creatinine ratio; NRF, normal renal function; NA, normal albuminuria; DRF, decreased renal function; EA, elevated albuminuria.

**Table 2:** Clinical characteristics of patients with T2D and different patterns of CKD.

Parameter	NRF/NA	DRF/NA	NRF/EA	DRF/EA
N	35	35	35	35
Age, years	64 (58–71)	65 (58–67)	64 (58–68)	66 (58–72)
Sex, m/f (n, %)	12/23 (34.3/65.7)	12/23 (34.3/65.7)	13/22 (37.1/62.9)	12/23 (34.3/65.7)
Body weight, kg	88 (75–98)	95 (80–104)	95 (80–108)	88 (73–100)
BMI, kg/m^2^	34.4 (28.1–38)	34.1 (30.1–37.5)	33.3 (30.8–39.6)	32.2 (28.8–36.2)
Obesity, n (%)	23 (65.7)	26 (74.3)	27 (77.1)	25 (71.4)
Waist circumference, cm	110 (99–130)	115 (106–123)	110 (105–124)	107 (104–112)
WHR	0.97 (0.93–1.10)	1.05 (0.90–1.10)	1.00 (0.93–1.09)	1.00 (0.91–1.07)
Current smokers, n (%)	3 (8.6)	4 (11.4)	5 (14.3)	3 (8.6)
Duration of T2D, years	12 (10–17)	13 (10–20)	14 (10–17)	14.5(11–18)
Insulin, n (%)	23 (65.7)	23 (65.7)	23 (65.7)	26 (74.8)
Metformin, n (%)	28 (80.0)	22 (62.8)	24 (68.6)	20 (57.1)
SU, n (%)	9 (25.7)	14 (40.8)	14 (40.0)	11 (31.4)
ACE inhibitors or ARBs, n (%)	24 (68.6)	29 (82.9)	28 (80.0)	30 (85.7)
Statins, n (%)	14 (40.0)	26 (74.3)	17 (48.6)	19 (54.3)
HbA_1C_, %	8.48 (7.5–10.3)	8.8 (7.88–9.81)	9.37 (8.17–11.8) ^§^	8.39 (7.46–9.53)
HbA_1C_, mmol/mol	69.2 (58.5–88.8)	72.7 (62.6–83.7)	78.9 (65.8–105) ^§^	68.1 (58–80.6)
CKD G1/G2/G3a/G3b, n (%)	10/25/0/0 (28.6/71.4/0/0)	0/0/23/12 (0/0/65.7/34.3)	3/32/0/0 (8.6/91.4/0/0)	0/0/23/12 (0/0/65.7/34.3)
eGFR, mL/min/1.73 m^2^	84 (71–94)	51 (45–55) ***	70 (65–78)	51 (43–54) ***
UACR, mg/mmol	0.6 (0.3–0.9)	0.65 (0.45–0.9)	10.5 (6.3–36.7) ^###^	12.0 (6.3–80.6) ^###^
CKD A1/A2/A3, n (%)	35/0/0 (100/0/0)	35/0/0 (100/0/0)	0/23/12 (0/65.7/34.3)	0/25/10 (0/71.4/28.6)
Protein excretion, g/day	0.07 (0.05–0.1)	0.07 (0.05–0.15)	0.17 (0.09–0.53) ^###^	0.2 (0.13–0.52) ^###^
Diabetic retinopathy, n (%)	19 (54.3)	19 (54.3)	23 (65.7)	24 (65.7)
Arterial hypertension, n (%)	33 (94.3)	35 (100)	34 (97.1)	35 (100)
Myocardial infarction in medical history, n (%)	4 (11.4)	7 (20.0)	5 (14.3)	11 (31.4)
Chronic heart failure NYHA class III/IV, n (%)	0 (0)	2 (5.7)	5 (14.3)	1 (2.9)
Cerebrovascular event in medical history, n (%)	1 (2.9)	7 (20.0)	3 (8.6)	3 (8.6)
Peripheral artery disease, n (%)	19 (54.3)	25 (71.4)	22 (62.9)	25 (71.4)

Continual parameters are presented as medians (interquartile range). Categorical data are presented as numbers (n) and percentage. ^§^ *p* < 0.05 vs. NRF/NA group (Mann-Whitney U-test), *** *p* < 0,001 vs. NRF/NA and DRF/NA groups, ^###^ *p* < 0.001 vs. NRF/NA and NRF/EA groups (Kruskal–Wallis H test); ACE, angiotensin-converting enzyme; ARBs, angiotensin II receptor blockers; BMI, body mass index; eGFR, estimated glomerular filtration rate; HbA_1C_, hemoglobin A_1C_; SU, sulfonylurea; T2D, type 2 diabetes; UACR, urinary albumin-to-creatinine ratio; WHR, waist-to-hip ratio.

**Table 3:** Factors associated with albuminuria in patients with T2D.

Parameter	Adjusted OR	95% CI	*p*-Value
Urinary RBP-4, μg/mmol	1.11	1.02–1.21	0.02
Urinary Col1, pg/mmol	1.16	1.01–1.32	0.03
Urinary HGF, ng/mmol	1.08	1.01–1.15	0.03

The results of logistic regression analysis. CI, confidence interval; OR, odds ratio. Parameters of the model with RBP-4: KS *p*-value < 0.001, area under ROC curve (AUC) = 0.71, sensitivity (Se) = 0.63, specificity (Sp) = 0.66for cut-off point L_P_ = 0.47, coefficients β: intercept (β_0_) = −3.09 (p>0.05), for RBP-4 = 0.10 (*p* = 0.02), for age = 0.023 (*p* > 0.05), for female sex = −0.019 (*p* > 0.05), for BMI = 0.0052 (*p* > 0.05), for duration of diabetes = −0.039 (*p* > 0.05), for HbA_1C_ = 0.16 (*p* > 0.05). Parameters of the model with Col1: KS *p*-value = 0.003, AUC = 0.71, Se = 0.63, Sp = 0.63for L_P_ = 0.473, coefficients β: β_0_ = −3.01 (*p* > 0.05), for Col1 = 0.15 (*p* = 0.03), for age = 0.025 (*p* > 0.05), for female sex = 0.059 (*p* > 0.05), for BMI = 0.00011 (*p* > 0.05), for duration of diabetes = −0.041 (*p* > 0.05), for HbA_1C_ = 0.18 (*p* > 0.05). Parameters of the model with HGF: KS *p*-value = 0.005,AUC = 0.67, Se = 0.64, Sp = 0.61 for L_P_ = 0.47; coefficients β: β_0_ = −3.34 (*p* > 0.05), for HGF = 0.075 (*p* = 0.03), for age = 0.016 (*p* > 0.05), for female sex = 0.15 (*p* > 0.05), for BMI = 0.011 (*p* > 0.05), for duration of diabetes = −0.034 (*p* > 0.05), for HbA_1C_ = 0.20 (*p* = 0.047).

**Table 4:** Factors associated with albuminuric CKD patternsin patients with T2D.

Parameter	Adjusted OR	95% CI	*p*-Value
NRF/EA			
Urinary RBP-4, μg/mmol	1.54	1.07–2.22	0.02
DRF/EA			
Urinary RBP-4, μg/mmol	1.36	1.02–1.83	0.04
Urinary Col1, pg/mmol	1.43	1.03–2.00	0.03

The results of logistic regression analysis with stepwise selection. CI, confidence interval; OR, odds ratio. Parameters of the model of NRF/EA: KS *p*-value < 0.001, area under ROC curve (AUC) = 0.78, sensitivity (Se) = 0.71, specificity (Sp) = 0.69for cut-off point L_P_ = 0.42; coefficients β: intercept (β_0_) = −1.47 (*p* > 0.05), for RBP-4 = 0.43 (*p* = 0.02), for age = −0.033 (*p* > 0.05), for female sex = 0.15 (*p* > 0.05), for BMI = 0.022 (*p* > 0.05), for duration of diabetes = −0.012 (*p* > 0.05), for HbA_1C_ = 0.20 (*p* > 0.05). Parameters of the model of DRF/EA with RBP-4: KS *p*-value = 0.003, AUC = 0.73, Se = 0.67, Sp = 0.69 for L_P_ = 0.42; coefficients β: β_0_ = −3.03 (*p* > 0.05), for RBP-4 = 0.31 (*p* = 0.04), for age = 0.037 (*p* > 0.05), for female sex = −0.26 (*p* > 0.05), for BMI = 0.0037 (*p* > 0.05), for duration of diabetes = −0.059 (*p* > 0.05), for HbA_1C_ = 0.044 (*p* > 0.05). Parameters of the model of DRF/EA with Col1: KS *p*-value = 0.02, AUC = 0.72, Se = 0.64, Sp = 0.71for L_P_ = 0.483; coefficients β: β_0_ = −3.01 (*p* > 0.05), for Col1 = 0.36 (*p* = 0.03), for age = 0.036 (*p* > 0.05), for female sex = 0.28 (*p* > 0.05), for BMI = −0.019 (*p* > 0.05), for duration of diabetes = −0.058 (*p* > 0.05), for HbA_1C_ = 0.14 (*p* > 0.05).

## Data Availability

The data presented in this study are available on request from the corresponding author.
